# Personality Stability From Age 14 to Age 77 Years

**DOI:** 10.1037/pag0000133

**Published:** 2016-12

**Authors:** Mathew A. Harris, Caroline E. Brett, Wendy Johnson, Ian J. Deary

**Affiliations:** 1 Centre for Cognitive Ageing & Cognitive Epidemiology and Centre for Clinical Brain Sciences, University of Edinburgh; 2 Centre for Cognitive Ageing and Cognitive Epidemiology, University of Edinburgh, and School of Natural Sciences and Psychology, Liverpool John Moores University; 3 Centre for Cognitive Ageing and Cognitive Epidemiology, University of Edinburgh

**Keywords:** personality, differential stability, aging, longitudinal study, 6-Day Sample

## Abstract

There is evidence for differential stability in personality trait differences, even over decades. The authors used data from a sample of the Scottish Mental Survey, 1947 to study personality stability from childhood to older age. The 6-Day Sample (*N* = 1,208) were rated on six personality characteristics by their teachers at around age 14. In 2012, the authors traced as many of these participants as possible and invited them to take part in a follow-up study. Those who agreed (*N* = 174) completed a questionnaire booklet at age 77 years, which included rating themselves and asking someone who knew them well to rate them on the same 6 characteristics on which they were rated in adolescence. Each set of 6 ratings was reduced to the same single underlying factor, denoted dependability, a trait comparable to conscientiousness. Participants’ and others’ older-age personality characteristic ratings were moderately correlated with each other, and with other measures of personality and wellbeing, but correlations suggested no significant stability of any of the 6 characteristics or their underlying factor, dependability, over the 63-year interval. However, a more complex model, controlling rater effects, indicated significant 63-year stability of 1 personality characteristic, Stability of Moods, and near-significant stability of another, Conscientiousness. Results suggest that lifelong differential stability of personality is generally quite low, but that some aspects of personality in older age may relate to personality in childhood.

“Personality refers to an individual’s characteristic patterns of thought, emotion, and behavior, together with the psychological mechanisms—hidden or not—behind those patterns” (Funder, 2013, p. 5). Individual differences in personality traits are associated with many important aspects of life, including job performance (Hurtz & Donovan, 2000), criminal behavior (Samuels et al., 2004), and health behaviors (Mõttus et al., 2013). Although personality traits show mean level change across the life course, there also appears to be substantial stability of individual differences in personality, even over several decades (Caspi & Roberts, 2001; Caspi, Roberts, & Shiner, 2005; Hampson & Goldberg, 2006; Roberts & DelVecchio, 2000). In this study, we assessed differential stability of personality across the life course, from adolescence (14 years) to older age (77 years).

Personality stability into older age has been observed before. For example, Leon, Gillum, Gillum, and Gouze (1979) followed a group of 281 middle-aged men who first completed the Minnesota Multiphasic Personality Inventory (MMPI) in 1947. Of the surviving members of the sample, 99 completed the MMPI again in 1977 at a mean age of 77 years. Correlations for each of the 13 personality scales between 1947 and 1977, across three decades of later adulthood, ranged from .28 to .74, indicative of moderate to strong stability of relative personality differences from middle to older age.

Such relative stability has also been observed over similar periods earlier in adulthood, and even from childhood. For example, Hampson and Goldberg (2006) followed a cohort of over 2000 Hawaiians, first studied as elementary schoolchildren (aged 6–12 years) between 1959 and 1967. At that time, participants were rated by their teachers on a number of attributes. Approximately 40 years later, 799 of the surviving members of the cohort completed questionnaires measuring the Big Five personality traits. Although trait stability correlations across later childhood and early adulthood were low for Neuroticism (.00) and Agreeableness (.08), modest correlations were apparent for Openness (.16), and particularly Conscientiousness (.25) and Extraversion (.29). Edmonds, Goldberg, Hampson, and Barckley (2013) reassessed stability in the same cohort using interviewer ratings in adulthood, which garnered higher stability correlations for Agreeableness (.19) and Openness (.24).

However, very few studies have assessed the relative stability of personality over longer periods. Soldz and Vaillant (1999) assessed stability across the majority of adulthood in a group of Harvard alumni, first assessed upon completing their studies at around age 22, then again at around age 67 years. The observed stability correlations for Neuroticism, Extraversion, and Openness from early to late adulthood were .20, .19, and .38, respectively. Further, Haan, Millsap, and Hartka (1986) observed stability correlations of .16 to .38 for six personality dimensions from early childhood (age 5–7 years) to late adulthood (age 55–62 years). However, as far as we are aware, there is no existing evidence of lifelong personality stability, both starting in early life, before adulthood, and extending into older age, after around 70 years of age. The study presented here assessed the relative stability from adolescence to older age of six single-item characteristic measures, devised by Terman (1925), and their underlying factor, dependability. Although dependability is not defined within the same reference frame as more widely known personality traits, it is perhaps most closely related to conscientiousness (Deary, Batty, Pattie, & Gale, 2008).

The construct of wellbeing is closely related to and perhaps even overlaps with personality, but also reflects maintenance of a life situation that fosters an ability to thrive (Weiss, Bates, & Luciano, 2008). Personality has at least been observed to correlate with contemporaneous wellbeing (DeNeve & Cooper, 1998; Schmutte & Ryff, 1997; Steel, Schmidt, & Shultz, 2008), and others have also shown personality to predict wellbeing over time. In particular, Friedman, Kern, and Reynolds (2010) found correlations between Neuroticism (−.29), Conscientiousness (.15) and Agreeableness (.15) in 1940 (at a mean age of 29 years), and subjective wellbeing 46 years later. Gale, Booth, Mõttus, Kuh, and Deary (2013) also assessed subjective wellbeing in participants aged 60 to 64 years, previously assessed on personality at ages 16 and 26 years. Each measure of wellbeing in later adulthood was positively associated with Extraversion and negatively associated with Neuroticism scores collected in youth (in each case a latent factor underlying measurements of the trait at both 16 and 26 years). Because of the association or overlap between personality and wellbeing, such results provide further insight into the longitudinal stability of personality. The present study therefore also assessed the relations between teachers’ ratings of personality characteristics in adolescence and measures of subjective wellbeing in the eighth decade of life.

Associations between personality and intelligence have been investigated for at least 100 years (Ackerman & Heggestad, 1997; Webb, 1915; Wechsler, 1950). Generally, socially desirable personality factors, such as Conscientiousness and Openness, are associated with higher cognitive performance, whereas traits such as Neuroticism and Psychoticism are negatively associated with intelligence (Ackerman & Heggestad, 1997; Chamorro-Premuzic, Furnham, & Ackerman, 2006; Schaie, Willis, & Caskie, 2004). However, these associations are generally quite weak and may change over time, becoming weaker in older age (Baker & Bichsel, 2006; Meier, Perrig-Chiello, & Perrig, 2002). Nonetheless, if intelligence is associated with personality, this should be considered when assessing the relative stability of personality. Intelligence was therefore also included in the present study.

We assessed the stability of personality differences and their relations with wellbeing and intelligence over more than six decades, from age 14 to age 77 years, thus spanning late childhood development and also extending well into what is generally considered to be older age. We made use of data from the 6-Day Sample, a representative sample of participants from the almost whole-year-of-birth Scottish Mental Survey, 1947 (Deary & Brett, 2015; Deary, Whalley, & Starr, 2009; Maxwell, 1969), who are described in further detail below. Childhood personality ratings on the 6-Day Sample have already been linked to later life outcomes. Deary et al. (2008) found that a measure of low adolescent dependability rated by teachers at about age 14 years—predicted mortality up to age 66 years, with a hazard ratio per standard deviation increase of .77 (95% confidence interval [CI]: .63, .94). This is comparable to other work predicting late-life outcomes from childhood personality assessments (Friedman et al., 1993, 1995a, 1995b). Although lifelong personality stability has not previously been assessed, existing evidence of personality stability from childhood to middle-late adulthood, and from early middle adulthood to older age suggests that personality shows some stability across the entire life course. We therefore expected to observe small to moderate stability of each of the six assessed personality characteristics, as well as their underlying factor, dependability, from later childhood right through to older age.

To frame this less widely known personality trait within the context of a more familiar and widely validated model of personality, we compared childhood and older-age dependability to older-age scores on the Big Five traits. We also assessed relations between dependability and measures of constructs related to personality, wellbeing and cognitive ability. We hypothesized that older-age wellbeing would also show substantial correlations with contemporaneous ratings of dependability, and a weak to moderate association with childhood dependability. Finally, we hypothesized at least a weak positive correlation between dependability and intelligence in childhood, but that this correlation might be even weaker in older age, and particularly over the interval of more than six decades.

## Method

### Participants

**Figure 1 fig1:**
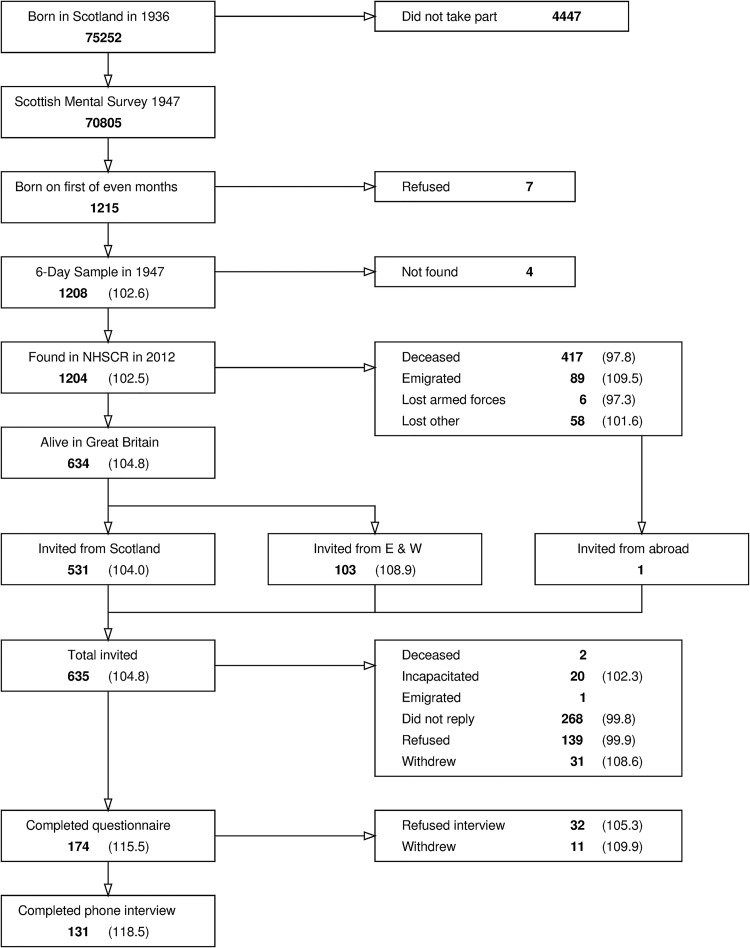
Participant selection flowchart. Mean IQ is included in brackets for each group of five or more members of the 6-Day Sample. Adapted from Deary and Brett (2015, Figure 1, p. 3).

The process of selection of the present study’s sample from the entire cohort of people born in Scotland in 1936 is shown in Figure 1 (adapted from Deary & Brett, 2015, Figure 1, p. 3). The majority of this cohort who were at school in Scotland on 4th June 1947 (mean age 10.9 years) took part in the Scottish Mental Survey, 1947, which consisted of a 45-min, group-administered intelligence test, the Moray House Test No. Twelve (Scottish Council for Research in Education [SCRE], 1933, 1949). The 1947 Scottish Mental Survey tested 70,805 children, that is, almost all Scottish schoolchildren born in 1936. A subsample of this cohort (*N* = 1,208) were selected for further study. As they were selected according to their date of birth being on one of 6 days of 1936—the 1st of February, April, June, August, October, and December—the sample became known as the *6-Day Sample*. This method of sampling also meant that the sample was representative of the cohort (MacPherson, 1958). These 1,208 original 6-Day Sample members were then studied more thoroughly, almost yearly, until the age of 27 (Deary et al., 2009). This began with an individually administered Stanford-Binet IQ test in 1947, followed by a series of home visits, during which data on a wide range of environmental factors were collected. In 1950 (mean age 13.9 years), these children’s teachers also rated them on six personality characteristics, described in further detail below.[Fig-anchor fig1]

In 2012, we traced as many of the original 6-Day Sample members as possible through the United Kingdom National Health Service Central Register. By 2012, 417 of the original sample members had died, another 89 had emigrated, and 68 could not be traced. The 635 who were found to be still alive and (with one exception) resident in Scotland, England, or Wales were invited to participate in a follow-up study (Brett & Deary, 2014; Deary & Brett, 2015). Most either declined to participate or did not respond to the invitation, but in 2013,^1^ those 174 individuals (92 female; mean age 76.7 years) who agreed to take part completed a detailed questionnaire booklet, which, among many other things, incorporated several questionnaires assessing personality and health. Participants who completed the questionnaire booklet were also asked to take part in a telephone interview, and 131 (72 female; mean age 77.1 years) agreed to do so. During the interview they completed a number of cognitive tests, based on sealed materials sent via post in advance (Deary & Brett, 2015). Surviving through to older age and agreeing to participate in the follow-up study was not random (Johnson, Brett, Calvin, & Deary, 2016). Those 174 who completed the questionnaire (and particularly those 131 who completed the telephone interview) had higher cognitive ability scores as children and were rated by teachers as more dependable, on average, than the population as a whole (Deary & Brett, 2015). Further details of participants included at each stage of the study are shown in Table 1 and further information on the follow-up subsample in relation to the full original 6-Day Sample can be found in Johnson et al. (2016).[Table-anchor tbl1]

### Measures

#### Personality characteristics in adolescence and older age

Around 1950, at about age 14 years, the 6-Day Sample members were rated on six personality characteristics by their teachers. These six characteristics—Self-Confidence, Perseverance, Stability of Moods, Conscientiousness, Originality, and Desire to Excel—were selected from the longer list of traits devised by Terman (1925) for his longitudinal study of over 1,500 gifted children. Teachers assessed their pupils on each characteristic using a single item rated on a 5-point scale, ranging from severely lacking to strongly displaying the characteristic. The six characteristics did not provide a comprehensive assessment of personality; in fact, the six items were quite correlated with one another (Self-Confidence: .01–.35; Perseverance: .11–.72; Stability of Moods: .01–.49; Conscientiousness: .01–.72; Originality: .08–.45; Desire to Excel: .21–.50), which allowed us to reduce them to a single dimension, which we termed *dependability* (as previously described; Deary et al., 2008). This factor only describes one aspect of personality, and not in terms of currently familiar personality models, such as the Big Five, which had not been articulated when our assessment was made. It appears to relate fairly closely to conscientiousness. As is usual in personality models, dependability did not explain all the variance in the six characteristics, but they did not provide enough information to identify additional factors.

Personality was assessed in older age using exactly the same measure as in childhood. At around age 77 years, those participants who completed the questionnaire booklet rated themselves on these same six items using the same rating scale, in relation to other people of roughly their current age. Participants were also requested to ask another person who knew them well (a spouse, friend or family member) to rate them on these characteristics in the same way. Including this additional set of ratings allowed us to assess self-other consistency, which could be taken as an index of validity of each personality measure; these correlations are reported in the Results section. All responses were converted to numerical values between 1 and 5.

#### International Personality Item Pool

Personality was also assessed at 77 years using the International Personality Item Pool (IPIP; Goldberg, 1999; Gow, Whiteman, Pattie, & Deary, 2005), which was also included in the questionnaire booklet. The IPIP provided measures of Extraversion, Agreeableness, Conscientiousness, Emotional Stability (the polar opposite of Neuroticism), and Intellect/Imagination (similar to Openness). The version used in this study comprised 50 items, including five subscales of 10 items measuring each trait. Each item was a first-person statement, for example, “I spend time reflecting on things.” Participants rated the accuracy of each statement, in relation to their own personalities, on a 5-point scale from 1 (*very inaccurate*) to 5 (*very accurate*). In the current sample, the five subscales showed internal consistencies of .84, .76, .74, .80, and .77, respectively.

#### Warwick-Edinburgh Mental Well Being Scale

Mental wellbeing was assessed using the Warwick-Edinburgh Mental Well Being Scale (WEMWBS; Tennant et al., 2007), another measure included in the questionnaire booklet. This is a 14-item questionnaire that asks people to report on their experiences of positive feelings; for example, “I’ve been feeling optimistic about the future” and “I’ve been feeling close to other people.” For each item, participants indicated how often they had felt that way, over the prior 2-week period, on a 5-point scale from 1 (*none of the time*) to 5 (*all of the time*). Tennant et al. provide an internal consistency of .89 and a test–retest reliability of .83 for the WEMWBS; we also measured internal consistency at .89 in the current sample.

#### Satisfaction With Life Scale

The five-item Satisfaction With Life Scale (SWLS; Diener, Emmons, Larsen, & Griffin, 1985), also included in the questionnaire booklet, was used as an additional measure of subjective wellbeing. Each item asked participants to indicate their agreement with a statement about their life satisfaction on a 7-point scale from 1 (*strongly agree*) to 7 (*strongly disagree*). Diener et al. (1985) reported that the SWLS has an internal consistency of .87 and a test–retest reliability of .82. In the current sample, we found the internal consistency to be .86.

#### Terman-Merrill Stanford-Binet Test

In 1947, at around age 11 years, all members of the 6-Day Sample completed Form L of Terman and Merrill’s (1937) revision of the Stanford-Binet Test, providing a measure of childhood intelligence (SB IQ; SCRE, 1947).

#### Moray House Test No. 12

Another measure of age-11 intelligence (MHT IQ) was derived from scores on the Moray House Test No. 12, completed by participants in the Scottish Mental Survey, 1947. The test is described and reproduced in full by the SCRE (1933). This is a group-administered test with a preponderance of verbal reasoning items. Whereas all subjects in the 6-Day sample had SB IQ scores, not all have MHT IQ scores, because 96 of the original 1,208 6-Day Sample participants (including 12 of those who completed the follow-up questionnaire) were absent from school on the day of the 1947 Scottish Mental Survey testing.

#### National Adult Reading Test

Before the telephone interview, testing materials were sent via post to participants in a sealed envelope. Verbal ability at age 77 years was measured using the National Adult Reading Test (NART; Nelson & Willison, 1991), which involves reading aloud a written list of 50 irregular words. During the interview, participants were instructed to open the sealed envelope, remove the list of words and read it aloud. The number of words pronounced correctly provided a measure of older-age verbal ability. As reported by Nelson and Willison, the NART shows high internal consistency (.91) and test–retest reliability (.98).

#### Raven’s Standard Progressive Matrices

A second cognitive test in older age, also administered by telephone, was Raven’s Standard Progressive Matrices (RSPM; Raven, 1938), a test of nonverbal reasoning. The RSPM comprises five sets of 12 progressively more difficult nonverbal problems, each of which asks participants to select the missing piece of a two-dimensional pattern from six (sets A and B) or eight (sets C to E) possible answer options. Participants also received the RSPM question booklet in the sealed envelope sent out before the telephone interview, and provided their answers verbally. The number of correct responses in 20 min provided a measure of fluid cognitive ability at age 77 years. Raven (1948) assessed the test–retest reliability of the RSPM in a range of age groups, providing estimates of up to .93 in young adults and .83 for those aged over 50 years.

#### Dementia

Participants who completed the questionnaire booklet provided details of their health, including whether or not they had been diagnosed with dementia.

### Analysis

Data were analyzed in SPSS Statistics 19, using the accompanying AMOS package for all statistical modeling. Three participants who reported having been diagnosed with dementia were excluded from all analyses due to the impact that dementia can have upon personality (Rubin, Morris, & Berg, 1987; Smith-Gamble et al., 2002). Other health factors may also impact on personality, but many of our participants reported at least some health problems and consistent impacts have rarely been suggested, so we only excluded those with dementia, most likely to affect personality due to neurodegeneration. Teachers’ ratings of adolescent characteristics were thought to be heavily influenced by intelligence (Maxwell, 1969) and were observed to be substantially correlated with the test scores, so were analyzed both with and without removing childhood IQ (the mean of the scores derived from each of the two tests taken at age 11). We used confirmatory factor analysis (CFA) for dimension reduction of personality characteristics, to assess construct continuity over time and across raters. Deary et al. (2008) previously subjected the 6-Day Sample’s adolescent personality characteristic ratings to principal component analysis, attributing much of the variability in the data to a component they denoted dependability. We therefore modeled the six characteristics as indicators of a single underlying factor, taken to represent the same trait.

This model was applied to the three sets of ratings—those provided by teachers (at age 14 years), and participants and others (at age 77 years)—first with all parameters allowed to be free, and subsequently with regression weights, intercepts, structural covariances, and residuals progressively constrained to be equal across the three rater groups. The final model, with residuals as well as all configural, metric, and scalar parameters constrained, tested strict invariance of the dependability factor across 63 years and different rater groups. We repeated this process, testing alternative two-factor models of the raw and residualized personality characteristics. For each two-factor model, we initially specified paths from individual characteristics to both underlying factors, except for the characteristic with the highest loading in the single-factor model, loading only on the first factor, and that with the lowest single-factor loading, loading only on the second factor. Weaker paths were then removed in turn until only six remained, with each characteristic loading on one underlying factor.

The latent variable scores estimated by the final constrained single-factor models were then used as our measure of dependability. We assessed personality stability by calculating Spearman’s coefficients of correlation between ordinal ratings of each characteristic provided at different times and by different raters. To adjust systematic rater bias, we residualized each individual characteristic over the latent dependability factor. We then repeated the longitudinal and interrater correlations using the residuals for each characteristic. We also accounted for participants who were not reassessed in older age by making full information maximum likelihood (FIML) estimates of the longitudinal stability correlations and older-age interrater correlations. We then calculated Pearson’s coefficients for correlations between dependability factors derived from the characteristic ratings provided by each rater, as well as for correlations between dependability and other measures of personality, wellbeing and intelligence. We calculated 95% CIs for each correlation and corrected for multiple comparisons according to the Holm-Bonferroni method (Holm, 1979).

As an alternative method of assessing stability of individual personality characteristics, while controlling systematic rater group effects, we tested a more complex model of all characteristic ratings. For each set of six ratings, we modeled an underlying rater group factor, intended to represent any systematic effects. We also modeled a latent variable representing each personality characteristic in older age, with loadings on corresponding self- and other-ratings constrained equal. Finally, the model included paths from teachers’ adolescent characteristic ratings to the older-age latent characteristics, representing the stability of each characteristic over the 63-year interval.

## Results

### Personality Construct Continuity

**Figure 2 fig2:**
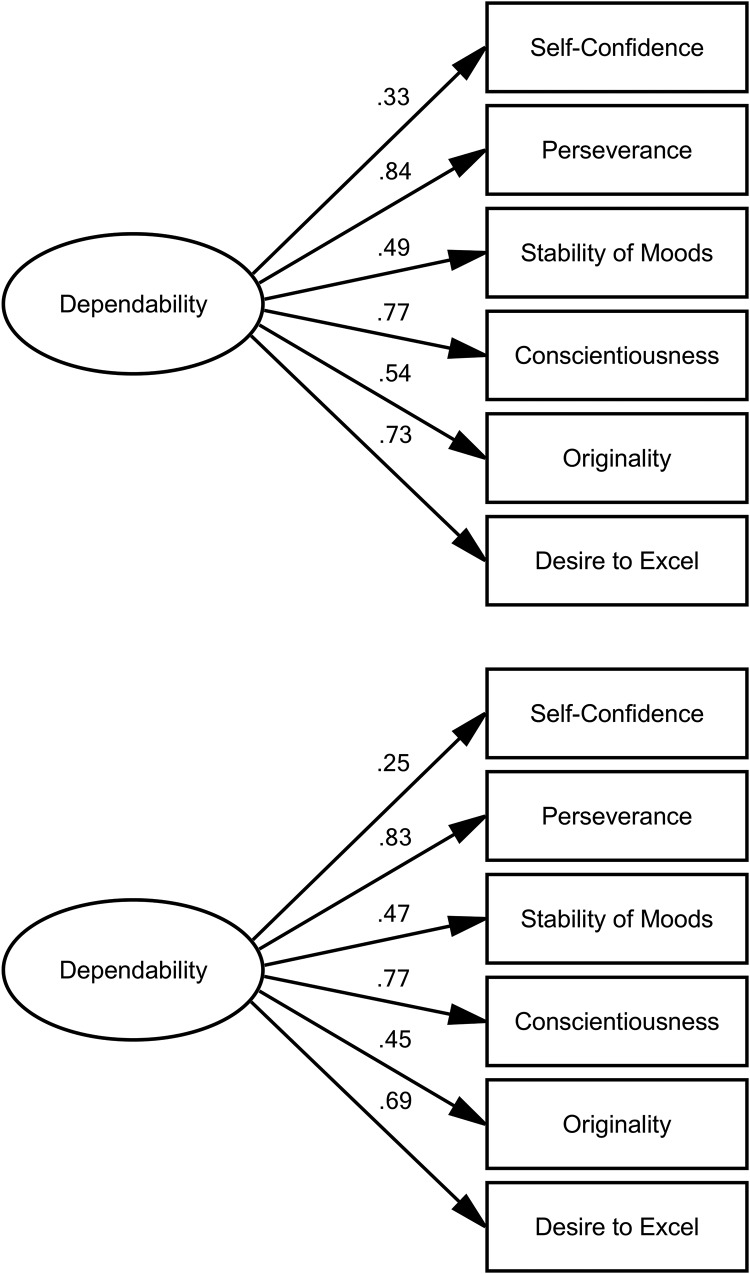
Confirmatory factor analysis results. Dependability was estimated as the single factor underlying ratings on all six characteristics, constraining regression weights, intercepts, structural covariances, and residuals equal across the three rater groups—teachers (at age 14 years), and participants and others (at age 77 years). Dependability was modeled both before (top) and after (bottom) removing IQ from teachers’ ratings. Path labels represent standardized regression coefficients.

We first ran the CFAs described above to evaluate the degree to which the ratings reflected a single common underlying construct across the long time span and different raters. The factor structure and regression weights are illustrated in Figure 2 and model fit statistics are presented in Table 2. As shown, the single-factor model did fit the data, although not perfectly (CFI = .84, RMSEA = .11), but constraining all parameters equal across rater groups, testing strict invariance of the dependability factor, did not, overall, impair model fit (CFI = 81, RMSEA = .08). Perseverance, Conscientiousness, and Desire to Excel were strongly associated with this factor, and Stability of Moods and Originality were moderately associated. These results were similar to the previous analyses of the 6-Day Sample’s adolescent characteristic ratings, which identified a dependability component on which Perseverance, Conscientiousness, and Stability of Moods loaded most heavily (Deary et al., 2008). Dependability therefore represents a trait describing motivation and diligence; an individual scoring high on this trait can be depended upon to get things done, to resist giving up, and perhaps to be more rational than emotional. On the other hand, confidence in oneself may facilitate this dependability but is less important.[Fig-anchor fig2][Table-anchor tbl2]

Repeating this analysis after removing childhood IQ from teacher ratings produced similar results, with only slightly lower estimated regression weights, although particularly for Self-Confidence and Originality (Figure 2). Again, the initial three-group model fit the data to an extent (CFI = .83, RMSEA = .11), and constraining all parameters equal across raters did not lead to an overall loss of fit (CFI = .79, RMSEA = .08). These results relate to CFAs that included older-age data (self and other ratings) for the 171 participants who participated in the follow-up and did not report having been diagnosed with dementia. The CFAs were repeated including self and other ratings from the three participants who did report a dementia diagnosis, and differences in the results were negligible.

We also tested alternative two-factor models of raw and residualized ratings, with Self-Confidence and Originality loading on the second factor. As reported in online supplementary materials (supplemental Figure S1; supplemental Table S1), these models did not fit the data as well as the single-factor models (raw ratings: CFI = .75, RMSEA = .09; residualised ratings: CFI = .74, RMSEA = .10). Scores on the latent variable estimated by the final constrained single-factor model were therefore taken to represent dependability and used as such in subsequent analyses.

### Longitudinal Personality Stability Correlations

We next investigated the associations between personality item characteristic ratings from adolescence, with and without controlling childhood IQ, and those provided approximately 63 years later, by the participants themselves and others who knew them. Correlations were assessed using Spearman’s coefficient for each individual personality characteristic, and Pearson’s coefficient for the underlying dependability factor extracted by CFA. The relations between older-age characteristic self-ratings and those made by others were analyzed in the same ways. All correlation coefficients are reported in Table 3. The strongest positive longitudinal correlation was between teachers’ and others’ ratings of Stability of Moods (*p* = .12, 95% CI [−.03, .27]; *p* = .11), but this did not achieve significance at α = .05, even without correcting for multiple comparisons and was weaker still (**p** = .10 [−.05, .24]; *p* = .20) for IQ-residualized teacher ratings. There was one significant longitudinal correlation, between residualized teachers’ ratings and others’ ratings of Desire to Excel (*p* = −.19, 95% CI [−.033, −.04]; *p* = .04), but this was a negative correlation and would not have remained significant after correcting for multiple comparisons. In contrast, correlations between participants’ self-ratings and others’ ratings in older age were moderate for all characteristics and dependability, ranging from .26 (CI [.11, .40]; *p* < .001) to .48 (CI [.35, .59]; *p* < .001). All were significant at α = .001, even after correcting for multiple comparisons.[Table-anchor tbl3]

We reran the stability correlations for teachers’, participants’ and others’ individual characteristic ratings, after adjusting for systematic rater bias. We did this by residualizing each of the individual six characteristics over dependability, which, as the factor underlying all six ratings, was bound to reflect any rater bias, as well as considerable content-relevant variance. This did not have substantial effects on the results (supplemental Table S2). All correlations between self-ratings and others’ ratings remained significant, ranging between .20 (CI [.12, .40]; *p* < .001) and .42 (CI [.29, .54]; *p* < .001). There were no significant longitudinal correlations, although the closest to achieving significance was between teachers’ and others’ ratings of Conscientiousness (*r* = .13 [−.02, .28]; *p* = .09). We repeated the correlations including the three participants with dementia, but again, this made negligible difference to the results. For all subsequent analyses, neither controlling rater effects nor excluding participants with dementia had a substantial effect on the results. To account for those participants assessed on the six personality characteristics in adolescence but not in older age, we also made FIML estimates of the stability correlations. These estimates did not differ substantially from the calculated correlation coefficients (supplemental Table S3).

### Longitudinal Model of Personality

**Figure 3 fig3:**
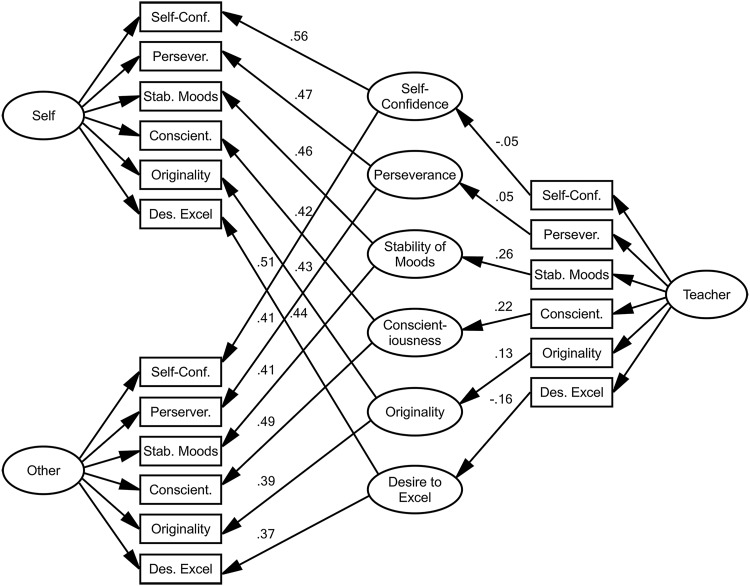
Longitudinal model of personality. Latent characteristic variables represent each personality characteristic in older age, with loadings on self- and other-ratings constrained equal. Latent rater group variables were included to control for systematic rater group effects. Path labels represent standardized regression coefficients. Rater group factor loadings and error terms are not shown for simplicity. Standardized regression weights of paths from childhood teacher-ratings to older-age latent characteristics were taken to represent the stability of personality from age 14 to age 77 years. Model fit statistics: comparative fit index = .83, root mean square error of approximation = .06.

We modeled all characteristic ratings together as an alternative method of assessing longitudinal stability of personality (Figure 3). For each set of ratings, we modeled an underlying rater group factor, designed to control any systematic effects, whether related to intelligence or other factors. Individual self- and other-ratings were modeled as indicators of six latent variables representing each of the assessed personality characteristics in older age. The standardized regression weights estimated for paths from each childhood teacher-rating to each corresponding older-age latent characteristic were taken to represent the 63-year stability of each characteristic. Under this model, teachers’ ratings directly reflected the corresponding latent characteristics, which in turn directly determined later self- and other-ratings; a rather strong set of assumptions about causal relations. The model fit the data fairly well (CFI = .826; RMSEA = .063; Table 4). As shown in Figure 3 and Table 4, the regression weights representing stability were −.05 (95% CI [−.26, .15]; *p* = .62) for Self-Confidence, .05 (CI [−.18, .28]; *p* = .66) for Perseverance, .26 (CI [.02, .50]; *p* = .03) for Stability of Moods, .22 (CI [−.01, .45]; *p* = .06) for Conscientiousness, .13 (CI [−.11, .37]; *p* = .30) for Originality, and −.16 (CI [−.41, .09]; *p* = .20) for Desire to Excel. For Stability of Moods and Conscientiousness, these estimates were above .2 (and significant or close to achieving significance at *p* < .05), indicating stability of these personality characteristics from childhood to older age.[Fig-anchor fig3][Table-anchor tbl4]

### Personality and Wellbeing

To compare the characteristic ratings to modern and better-validated measures of personality, we calculated the correlations between the dependability factor derived from each set of ratings and scores on the five scales measured by the IPIP. Furthermore, as four of the Big Five personality traits have been shown to relate to wellbeing, we also assessed correlations between dependability and scores on the WEMWBS and SWLS. As shown in Table 5, dependability in older age, based on participants’ self-ratings, showed moderate positive correlations with each of the five IPIP scales, ranging from .24 (CI [.10, .38]; *p* < .01) for Emotional Stability to .53 (CI [.41, .63]; *p* < .001) for Conscientiousness. Correlations between self-rated dependability in older age and measures of wellbeing were also moderate, at .54 (CI [.42, .64]; *p* < .001) and .38 (CI [.24, .50]; *p* < .001). All these correlations were significant after correcting for multiple comparisons. Dependability derived from others’ ratings also showed moderate positive correlations with all five IPIP scales and both wellbeing measures, ranging from .20 (CI [.05, .34]; *p* = .01) for Emotional Stability to .36 (CI [.22, .48]; *p* < .001) for Conscientiousness. Correlations between teacher-rated dependability and older-age measures of personality and wellbeing were much weaker, ranging between −.17 (CI [−.31, −.02]; *p* = .03) for Conscientiousness (in the opposite direction to that which we had predicted) and .17 (CI [.02, .31]; *p* = .03) for Emotional Stability. These were the only two results that achieved significance at α = .05; neither was still significant when dependability was derived from residualized teacher ratings, and nor did they remain significant after correcting for multiple comparisons.[Table-anchor tbl5]

### Personality and Intelligence

Finally, we tested the relations between personality and intelligence over 66 years by calculating the correlations among adolescent and older-age dependability, two measures of childhood IQ, and performance on two cognitive tests in older age. These results are included in Table 5. Contemporaneously, adolescent dependability correlated with SB IQ (*r* = .39 [.34, .44]; *p* < .001) and MHT IQ (*r* = .38 [.33, .43]; *p* < .001). Older-age self-rated dependability only correlated weakly with scores on the NART (*r* = .09 [−.08, .26], *p* = .31) and RSPM (*r* = .08 [−.10, .25], *p* = .40), but for older-age dependability as rated by others, the correlation with NART performance was slightly stronger (*r* = .17 [−.00, .34], *p* = .05) and that with RSPM performance was stronger still (*r* = .29 [.12, .44], *p* < .001). Longitudinally, there was no significant correlation between adolescent dependability and performance on the RSPM (*r* = .16 [−.02, .32], *p* = .08), nor between either SB IQ (*r* = .04 [−.11, .19], *p* = .61) or MHT IQ (*r* = .08 [−.08, .23], *p* = .34) and older-age dependability, as derived from self-ratings. However, both measures of childhood IQ predicted older-age dependability when rated by others (SB IQ: *r* = .23 [.08, .37], *p* < .01; MHT IQ: *r* = .18 [.03, .33], *p* = .02). Childhood dependability also predicted performance on the NART (*r* = .20 [.03, .36], *p* = .02), although these correlations did not remain significant after correcting for multiple comparisons.

## Discussion

The main objective of this study was to assess the differential stability of personality characteristics from late childhood through to older age, in the eighth decade of life. Our participants were rated on the same characteristics at around age 14 and again at around age 77, making this the longest-spanning study of personality stability of which we are aware. We found that personality characteristic ratings provided by teachers in adolescence, and by participants and others in older age, all showed a similar underlying structure, with each set able to be reduced to a single factor we termed dependability. Contrary to our hypotheses, there were no positive correlations strong enough to achieve significance between adolescent and older-age characteristic ratings or dependability. However, a more complex model, controlling rater effects, estimated longitudinal stability effect sizes of more than .2 for two characteristics, Stability of Moods and Conscientiousness. Older-age self- and other-rated dependability was, as expected, correlated with the five other personality scales, particularly Conscientiousness, and both wellbeing measures, although we had also expected adolescent dependability to relate to these later-life measures, and it did not. We did find some evidence indicating that personality and intelligence are related throughout the life span; childhood IQ was correlated with dependability in adolescence, and also predicted dependability in older age when it was rated by others. Older-age cognitive ability also showed weak associations with dependability in older age and in adolescence.

Previous studies have demonstrated relative stability of personality, even over as much as 40 or 50 years, from childhood to middle age (Haan et al., 1986; Hampson & Goldberg, 2006; Edmonds et al., 2013), early to late adulthood (Soldz & Vaillant, 1999), and from middle age to older age (Leon et al., 1979). We hypothesized that we would find evidence of personality stability over an even longer period of 63 years, but our correlations did not support this hypothesis, appearing inconsistent with previous results. One major difference between our study and most previous studies is that our participants’ later-life characteristic ratings were provided in older age, rather than middle to late adulthood. The lack of evidence for personality stability in our study may be related to the impact of changes in life circumstances, and declines in physical and cognitive abilities common in older age (Weiss et al., 2005). However, previous research suggests that personality is relatively stable over short periods in older age (Mõttus, Johnson, & Deary, 2012), and even over 30 years from middle age to old age (Leon et al., 1979).

Our study differed from that of Leon et al. (1979) in assessing stability from late childhood. Childhood is a period of intense learning and many new experiences, leading to much more frequent small changes in personality, or much more substantial changes over time (Caspi & Roberts, 2001; Glenn, 1980). Even over relatively short periods, test-retest correlations are much weaker in childhood than in later life (Caspi et al., 2005; Hampson & Goldberg, 2006; Mõttus et al., 2012; Roberts & DelVecchio, 2000). Adolescence is a particularly dynamic period of personality development, as individuals tend to become more mature as they enter adulthood (Blonigen, Carlson, Hicks, Krueger, & Iacono, 2008; Johnson, Hicks, McGue, & Iacono, 2007; Roberts, Caspi, & Moffitt, 2001). As our participants were first rated at an average age of around 14, subsequent maturational development likely contributed substantially to the incongruence between adolescent and older age characteristic ratings. However, prior studies assessed personality stability from childhood and would therefore have been affected in the same way, yet they still showed evidence of greater stability.

Although previous studies have assessed personality stability either from childhood or from some time in younger adulthood into older age, to our knowledge, no previous studies have assessed stability over such a long interval, incorporating both periods of more rapid change in personality. Furthermore, it is generally recognized that personality continues to change throughout life, and stability correlations tend to be weaker over longer intervals (Caspi et al., 2005; Roberts & DelVecchio, 2000). Although our correlational results initially appear inconsistent with previous findings of personality stability over several decades, these previous studies did not by any means demonstrate perfect stability, and therefore also provide evidence that personality changes. Our findings and previous findings, taken together, provide support for the hypothesis that personality changes gradually throughout life, which can lead to personality in older age being quite different from personality in childhood.

Although the correlations did not provide evidence of personality stability from adolescence to older age, a more complex model did indicate some stability of two characteristics, Stability of Moods and Conscientiousness. This model included a latent variable for each set of ratings, which should have controlled systematic rater group effects better than simply removing IQ from the teacher-ratings. It also combined older-age self- and other-ratings, thereby likely representing a better estimate of each assessed characteristic in later life. At the same time, however, it required rather strong assumptions regarding causal relations among teacher ratings, and later self- and other-ratings of their characteristics. This set of assumptions may not have been met but would tend to increase the likelihood of perceiving stability. Nonetheless, at least four of the six characteristics still showed no clear evidence of stability from age 14 to age 77 years, consistent with the results of the correlations, and with a conclusion that personality in older age may be quite different from personality in childhood.

However, Stability of Moods and Conscientiousness did show moderate 63-year stability under this model, indicating that older-age personality and childhood personality may not be completely unrelated. The higher stabilities of these two characteristics in particular were also consistent with previous research showing stability of related personality traits, neuroticism/emotional stability (Soldz & Vaillant, 1999) and particularly conscientiousness (Edmonds et al., 2013; Hampson & Goldberg, 2006). Interestingly, residualizing teacher-rated Stability of Moods reduced its correlation with older-age self-rated Stability of Moods to almost zero, suggesting that intelligence played a role in the stability of this characteristic observed in the more complex model. For example, to the extent it was stable, higher intelligence may have led to higher actual Stability of Moods through better ability to manage environmental circumstances to advantage, and could serve as a resource for emotional stability in older age.

Further, we did find that the structure of personality was fairly similar across the 63-year interval and three rater groups. We were able to assess this by CFA as, in contrast to many prior studies of long-term personality stability, we used the same measures in older-age that were used in adolescence. Each set of six characteristic ratings was reduced to the same single underlying factor by CFA, each showing fairly good fit, and better fit than an alternative two-factor model. Further, all parameters of the model could be constrained equal across the three sets without a marked loss of fit. This invariance also indicated validity of dependability as a personality trait. However, the fit of each model was not perfect, because not all characteristics were similarly closely related to dependability. Other aspects of personality must have influenced variation in these characteristics, but from the data provided by these six characteristics alone, we could not reliably derive any additional underlying factors.

Further, differences across raters could have manifested in the variation of the actual data from the specified model. In particular, teacher ratings were more closely related to intelligence, while residualising these ratings likely removed some valid shared variance with intelligence. Regression weights for Self-Confidence and Originality were reduced by almost .1, suggesting that these characteristics were either particularly related to intelligence in childhood, or particularly susceptible to the influence of intelligence on teachers’ ratings. As raw ratings included the erroneous influence of intelligence on teacher-ratings, although residualized ratings may have been missing some valid shared variance with intelligence, neither derived measure of childhood dependability was likely to be exactly the same factor as those derived in older age. The fact that the underlying structure may have varied slightly across time and rater groups does limit the interpretation of our results on the stability of dependability. In addition, the individual characteristics varied in longitudinal stability, which may have reduced the stability of their common factor.

As dependability is not a commonly used measure of personality, we also assessed its relation to the Big Five personality traits. Whether derived from self-ratings or from others’ ratings, dependability was at least modestly related to all five of these other measures of personality in older age. Although this indicated that dependability describes an aspect of personality using a different frame of reference to the five-factor model, it also demonstrated that it can be represented in terms of these five factors, and fits within its nomological network. Older-age dependability also correlated with both measures of wellbeing, consistent with our hypothesis and with previous work demonstrating close relations between personality and wellbeing (DeNeve & Cooper, 1998; Schmutte & Ryffe, 1997; Steel et al., 2008). We had also hypothesized that adolescent dependability would predict measures of older-age wellbeing, but this hypothesis was not supported. However, in light of our finding that adolescent dependability did not significantly predict older-age dependability, it would not necessarily be expected to predict wellbeing either.

Our results did indicate an association between personality and intelligence over 66 years. Teachers’ characteristic ratings were related to IQ in childhood, and others’ ratings were related to cognitive ability in older age. Adolescent dependability also predicted NART performance in older age, whereas childhood IQ predicted older-age dependability (when derived from others’ ratings). However, these longitudinal associations reflected the stability of intelligence, previously demonstrated in the 6-Day Sample (Deary & Brett, 2015) and the influence of intelligence on teachers’ and others’ ratings of personality. Childhood dependability no longer predicted older-age intelligence when controlling childhood IQ, and childhood IQ no longer predicted later dependability when controlling older-age intelligence.

In fact, the convolution of teachers’ and others’ characteristic ratings with contemporaneous measures of intelligence was a limitation of this study. In contrast to subjective ratings, any observer ratings of personality are necessarily based on social interaction and observable behavior, which is guided not only by personality, but also by cognitive ability. For example, more intelligent individuals should be better able to understand and thereby to interact with others, as well as better able to understand and know how to behave appropriately in different situations. This illustrates how observers may be influenced by intelligence when rating personality, an important consideration for future studies of personality and intelligence. Observers could also be influenced by a number of other irrelevant factors, such as appearance, their opinion of a subject’s family, or prejudices regarding the area in which a subject lives.

Of course, there were likely differences among individual raters within each group in how much intelligence (or any of a number of other factors) influenced their personality ratings. This was demonstrated by the correlations between self- and other-ratings in older age, which ranged from .26 to .48. These correlations may seem low for assessments of the same constructs, but self- and other-ratings of personality are known to diverge, sometimes substantially. For example, Watson, Hubbard, and Wiese (2000) observed self-other correlations for ratings on the Big Five traits of .34 to .48 among friends and .49 to .61 among married couples, and lower correlations for other personality traits. Vazire (2010) observed correlations of .20 to .54 participants’ and their friends’ ratings of facets of neuroticism, extraversion and intellect. Our results were comparable, or perhaps a little lower, but this may be related to personality change in older age.

Rater bias may have been more consistent among teachers, who would have all had more similar relationships with the participants to one another. The teachers’ ratings within their context may make this up in the form of less measurement error, as the varied circumstances and relationships to the subject in more general groups of others’ ratings introduce considerable additional sources of measurement error. Nonetheless, Laidra, Allik, Harro, Merenäkk, and Harro (2006), who assessed personality ratings completed by a group of over 500 adolescents, their parents and their teachers, found that teachers’ ratings were least closely related to those of other raters. This may have been due to a relatively consistent bias among all teachers; a halo effect that could be expected to lead teachers to rate pupils who performed better academically as higher on the six personality characteristics as well. Previous evidence does suggest that halo effects influence teachers’ opinions of their pupils (Abikoff, Courtney, Pelham, & Koplewicz, 1993) and, more specifically, that teachers’ personality ratings are related to academic achievement (Scandette & Richter, 1971).

Among the 6-Day Sample, teacher ratings were indeed moderately correlated with childhood IQ, a bias we attempted to control by residualizing teacher ratings over IQ. This still may not have made their ratings any more comparable to those provided by participants themselves and their friends and relatives, because each rater type is subject to different kinds of bias. However, the Hawaii cohort studies discussed above also used teacher ratings of personality in childhood, and found that they were predictive of self-ratings (Hampson & Goldberg, 2006) and peer ratings (Edmonds et al., 2013) of personality in middle age. Similarly, Pulkkinen, Kokko, and Rantanen (2012) were able to predict personality at age 42 from teacher ratings in childhood and adolescence. Furthermore, in contrast to the findings of Laidra et al. (2006); Baker, Victor, Chambers, and Halverson (2004) found that, in a sample of 165 students of a small high school, teacher ratings of the Big Five personality factors were particularly reliable in comparison to self and peer ratings.

Finally, our more complex model, which should have better controlled for systematic effects of rater group, did indicate some possible stability of Conscientiousness and Stability of Moods. Although the model required rather strong causal assumptions, for which independent evidence would need to be developed, the results suggested that rater effects could have distorted the correlational results for these characteristics. However, the stability estimates for these two characteristics were still fairly low, whereas the estimated stabilities of the other four characteristics were even lower, suggesting that weakness of the longitudinal correlations could not be attributed solely to rater bias.

This study was limited by the representativeness of the follow-up sample. Although the original 6-Day Sample included 1,208 participants, almost perfectly representative of the entire cohort of Scottish people born in 1936, not all of the original participants were able and willing to take part in the follow-up study. However, the follow-up sample was less representative, as inclusion depended on the original members having survived to age 77 years, and being both able and willing to participate. Those who were still alive and who volunteered to participate were, on average, more intelligent and more dependable (Johnson et al., 2016). Variances in childhood IQ and dependability in our follow-up sample were reduced to 53% and 82%, respectively, of the original variances in the entire sample. This was not specific to the 6-Day Sample follow-up study’s recruitment or assessment methods, as variance in relations to mortality would be similarly subject to effect by restriction of population-representativeness of sample in this age group. However, the reduction in variance may have led to underestimation of the stability of personality characteristics.

A related limitation was that the follow-up sample of 171 participants may have been too small to detect any significant stability of personality, as some of the correlations observed would have achieved significance if observed in a larger sample. However, this sample size still provided enough power to detect small correlations of around .2, and a larger sample would not of course be expected to increase the strength of the correlations. Regardless of significance, the relative stability correlations we observed were much weaker than those of previous studies of personality stability over shorter periods. The sample size, however, had very low power to assess measurement invariance. This was compounded by the rather poor fits of the models. Thus, the conclusion that dependability was measured in the same way across the 63 years should be considered tentative.

One further limitation of the study is the number of items used to assess personality both in adolescence and in older age. Each characteristic was measured by a single item, meaning that the underlying factor, dependability, was derived from only six items. Single-item assessments of personality are sometimes useful, particularly when personality is not the primary focus of a study, and therefore does not warrant more extensive assessment (as was the case in the original 6-Day Sample study). Furthermore, Woods and Hampson (2005) assessed the test-retest reliabilities of their single-item measures of personality (SIMPs) over periods of between a month and a year, observing mean correlations of between .060 for agreeableness and .78 for extraversion. Spörrle and Bekk (2014) observed slightly higher mean reliabilities of between .71 for agreeableness and .81 for extraversion using the same SIMPs translated into German. However, the short-term stability of ratings on the characteristics used in the present study has not been assessed, and it is unlikely to be as high as that of multi-item measures of more established personality factors. The reliability of the assessed characteristics may also be lower in older age, particularly if aging-related cognitive and physical impairments affect individuals’ abilities to provide accurate ratings, but again, this has not been established. It is possible that we may have found more evidence for personality stability had the original assessment of adolescent personality been more extensive. Finally, we had access to assessments of personality at only two time points; including additional intermediary assessments would help future studies identify how personality changes throughout life. With personality assessments at additional time points, lifelong change could be assessed using nonlinear trajectories, which may be more appropriate.

In this study, we assessed the stability of personality over an interval of 63 years, from adolescence through to older age. We observed that individual differences in personality characteristics in later life were not closely related to the same traits in early life. Controlling rater-group effects did reveal marginally significant and near-significant stability of two of the six assessed personality characteristics at the expense of imposing other assumptions, but results generally indicated very low stability of personality from age 14 to age 77 years. Previous studies have demonstrated that personality is subject to a lifelong series of relatively small changes—particularly in adolescence and early adulthood, but continuing even into older age. As a result of this gradual change, personality can appear relatively stable over short intervals—increasingly so throughout adulthood. However, the longer the interval between two assessments of personality, the weaker the relationship between the two tends to be. Our results suggest that, when the interval is increased to as much as 63 years, there is hardly any relationship at all. If so, personality changes only gradually throughout life, but by older age it may be quite different from personality in childhood. Future studies should focus on developing better understanding of how and why personality changes throughout the life course.

## Supplementary Material

10.1037/pag0000133.supp

## Figures and Tables

**Table 1 tbl1:** Descriptive Statistics of the 6-Day Sample Studied in Childhood and in Older Age

Characteristic	1947		1950		2013a		2013b	
	*M*	*SD*	*M*	*SD*	*M*	*SD*	*M*	*SD*
*N*	1,208		1,198		171		129	
Gender (M/F)	590/618		585/613		82/89		59/70	
Age	10.9	.3	13.9	.6	76.7	.4	77.1	.3
SB IQ	102.6	20.1	102.6	20.7	115.7	19.7	118.7	19.1
MHT IQ	100.0	15.0	100.0	15.0	110.1	11.2	111.2	10.9
Self-Confidence			3.0	.8	3.2	.8	3.3	.7
Perseverance			2.9	.9	3.3	.8	3.4	.8
Stability of Moods			3.3	.9	3.6	1.0	3.6	1.0
Conscientiousness			3.2	.9	3.4	.9	3.5	.9
Originality			2.6	.8	3.0	.8	3.0	.7
Desire to Excel			2.9	.9	3.2	.8	3.3	.8
WEMWBS score					54.6	7.0	55.2	6.4
SWLS score					27.1	.4	27.0	.5
NART score							35.1	8.0
RSPM score							33.7	7.5
*Note.* SB = Stanford-Binet; IQ = intelligence quotient; MHT = Moray House Test; WEMWBS = Warwick Edinburgh Mental Well-Being Scale; SWLS = Satisfaction With Life Scale; NART = National Adult Reading Test; RSPM = Raven’s Standard Progressive Matrices. Sample summaries are provided for the subset of participants included at each of four stages: childhood intelligence testing (in 1947), adolescent characteristic rating (in 1950), follow-up questionnaire completion (in 2013a) and older-age intelligence testing (in 2013b). Participants with dementia were excluded from the two later subsets summarised in this table. The personality item ratings summarised for each subset are those provided by teachers during adolescence.								

**Table 2 tbl2:** Confirmatory Factor Analysis Model Fit Statistics

Model	χ^2^	*df*	*p*	χ^2^/*df*	NFI	CFI	RMSEA
Raw ratings							
Unconstrained	551.27	27	<.001	20.42	.83	.84	.11
Constrained	666.91	63	<.001	10.59	.80	.81	.08
Excluding IQ							
Unconstrained	505.55	27	<.001	18.72	.82	.83	.11
Constrained	638.13	63	<.001	10.13	.78	.79	.08
*Note.* NFI = normed fit index; CFI = comparative fit index; RMSEA = root mean square error of approximation. Dependability was estimated as the single factor underlying ratings on all six characteristics, constraining regression weights, intercepts, structural covariances and residuals equal across the three rater groups: teachers (at age 14 years), and participants and others (at age 77 years). Dependability was modelled both before and after removing IQ from teachers’ ratings.							

**Table 3 tbl3:** Personality Stability Correlations

Characteristic	Teacher (14 years) versus		Teacher (14 years; ex. IQ) versus		Self (77 years) versus Other (77 years)
	Self (77 years)	Other (77 years)	Self (77 years)	Other (77 years)	
Self-confidence	.02	.01	−.02	−.05	.48
Perseverance	−.05	−.03	−.06	−.07	.44
Stability of moods	.12	.12	.01	.10	.26
Conscientiousness	−.01	.12	.00	.06	.39
Originality	.11	.04	.08	−.06	.35
Desire to excel	−.01	−.14	−.02	−.19	.41
Dependability (factor)	−.04	−.02	−.07	−.11	.48
*Note.* Spearman’s ρ coefficients are reported for correlations reflecting the stability of each individually rated personality characteristic. Pearson’s *r* coefficients are reported for correlations between the derived dependability factors.					

**Table 4 tbl4:** Longitudinal Model of Personality Fit Statistics and Characteristic Stability Estimates

	β		*SE*		*p*	
Self-confidence	−.05		.10		.62	
Perseverance	.05		.12		.66	
Stability of moods	.26		.12		.03	
Conscientiousness	.22		.12		.06	
Originality	.13		.12		.30	
Desire to excel	−.16		.13		.20	
χ^2^	*df*	*p*	χ^2^/*df*	NFI	CFI	RMSEA
713.57	123	<.001	5.80	.80	.83	.06
*Note*. Standardized β coefficients are reported for correlations reflecting the stability of each individually rated personality characteristic.						

**Table 5 tbl5:** Correlations Between Dependability Factors in Adolescence and Older Age, and Personality (Older Age), Wellbeing (Older Age), and Intelligence (Childhood and Older Age)

Characteristic	Teacher (14 years)	Teacher (ex. IQ)	Self (77 years)	Other (77 years)
Extraversion	.01	−.09	.39	.32
Agreeableness	.06	.03	.25	.36
Conscientiousness	−.17	−.13	.53	.36
Emotional stability	.17	.05	.24	.20
Intellect/imagination	.06	−.11	.41	.33
Mental wellbeing	.00	−.06	.54	.34
Satisfaction with life	−.08	−.11	.38	.20
SB IQ	.39		.04	.23
MHT IQ	.38		.08	.18
NART score	.20	−.07	.09	.17
RSPM score	.16	−.04	.08	.29
*Note.* SB = Stanford-Binet; IQ = intelligence quotient; MHT = Moray House Test; NART = National Adult Reading Test; RSPM = Raven’s Standard Progressive Matrices. Pearson’s *r* coefficients are reported for all correlations between dependability factors and other measures of personality, wellbeing, and intelligence.				

## Footnotes

1 Some participants completed the questionnaire booklet in late 2012.
