# Supplementary material for: Personality Stability From Age 14 to Age 77 Years
Source: Psychol Aging. 2016 Dec;31(8):862–74. doi: 10.1037/pag0000133 (PMC5144810; doi:10.1037/pag0000133)
Supplement: Supplementary file 1 [file PAG-2015-0437_Harris_Supp_Mat.docx]

Figure S1

*Two-factor confirmatory factor analysis results*

*
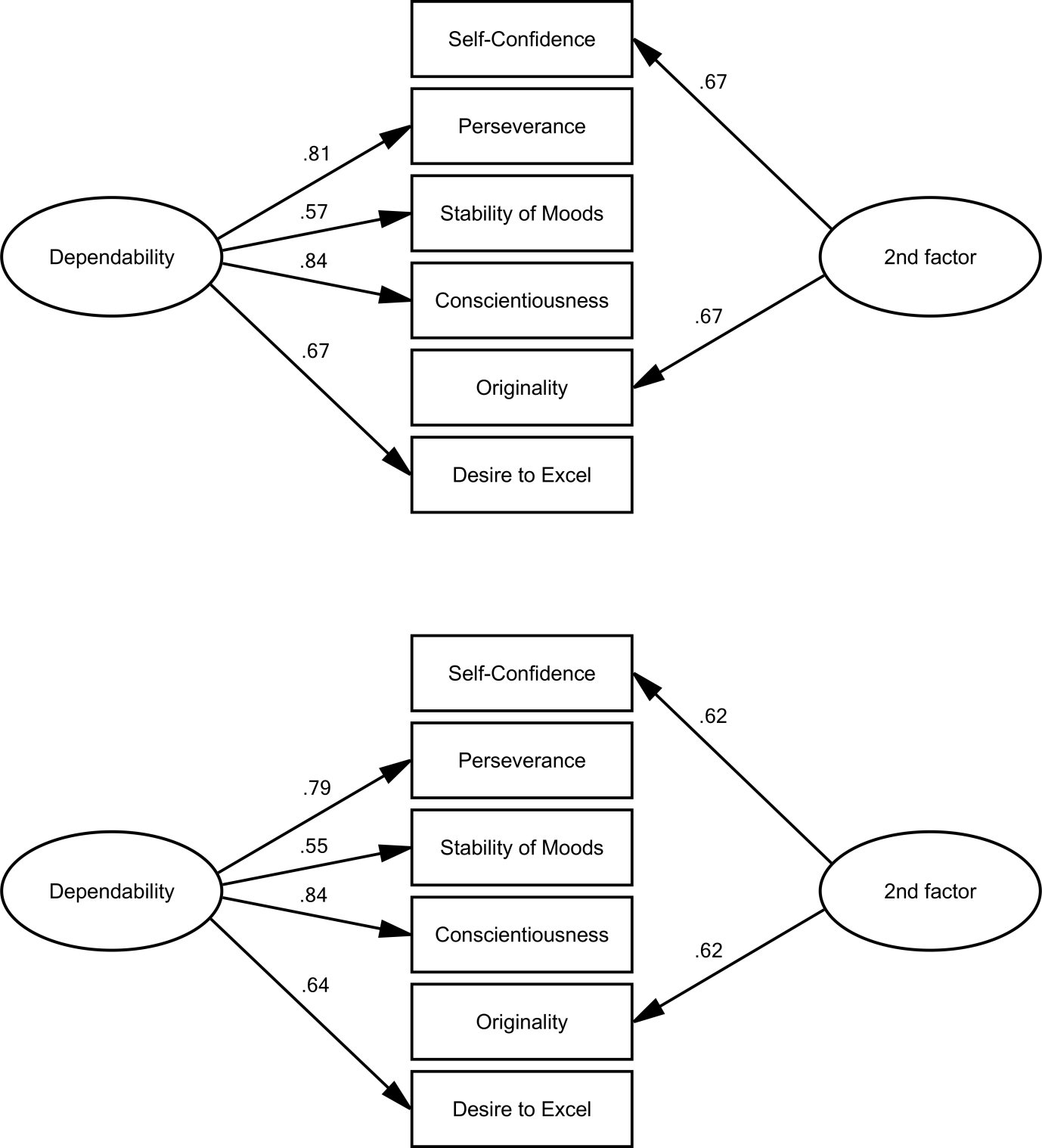
*

*Note.* Two-factor model structures, regression weights, intercepts, structural covariances and residuals were constrained equal across the three rater groups – teachers (at age 14 years), and participants and others (at age 77 years). These two factors were modelled both before (*top*) and after (*bottom*) removing IQ from teachers’ ratings. Path labels represent standardised regression coefficients.

Table S1

*Two-factor confirmatory factor analysis model fit statistics*

|  | χ^2^ | df | p | χ^2^/df | NFI | CFI | RMSEA |
| --- | --- | --- | --- | --- | --- | --- | --- |
| Raw ratings | 748.98 | 60 | <.001 | 12.48 | .74 | .75 | .09 |
| Residualised | 894.76 | 60 | <.001 | 14.91 | .73 | .74 | .10 |

*Note.* Two-factor model structures, regression weights, intercepts, structural covariances and residuals were constrained equal across the three rater groups – teachers (at age 14 years), and participants and others (at age 77 years). Dependability was modelled using both before and after removing IQ from teachers’ ratings.

Table S2

*Personality stability correlations, controlling rater effects*

|  | Teacher (14yrs) versus | | Self (77yrs) versus |
| --- | --- | --- | --- |
|  | Self (77yrs) | Other (77yrs) | Other (77yrs) |
| Self-Confidence | .01 | -.04 | .42 |
| Perseverance | .08 | .04 | .35 |
| Stability of Moods | .07 | .07 | .28 |
| Conscientiousness | .11 | .13 | .27 |
| Originality | .11 | .10 | .28 |
| Desire to Excel | .07 | .00 | .32 |

*Note.* Pearson’s *r* coefficients are reported for correlations reflecting the stability of each individually rated personality characteristic, controlling dependability, the factor underlying all six characteristics.

Table S3

*Full information maximum likelihood estimates of personality stability correlations*

|  | Teacher (14yrs)  versus | | Teacher (14yrs; ex. IQ)  versus | | Self (77yrs)  versus |
| --- | --- | --- | --- | --- | --- |
|  | Self (77yrs) | Other (77yrs) | Self (77yrs) | Other (77yrs) | Other (77yrs) |
| Self-Confidence | .01 | .01 | -.03 | -.05 | .48 |
| Perseverance | -.04 | -.03 | -.05 | -.07 | .45 |
| Stability of Moods | .11 | .13 | .09 | .11 | .27 |
| Conscientiousness | .01 | .12 | -.01 | .06 | .39 |
| Originality | .14 | .05 | .11 | -.05 | .36 |
| Desire to Excel | -.01 | -.14 | -.02 | -.19 | .41 |
| Dependability (factor) | -.04 | -.02 | -.07 | -.11 | .48 |

*Note.* Full information maximum likelihood (FIML) estimates are reported for correlations reflecting the stability of each individually rated personality characteristic, as well as the dependability factor underlying all six characteristics.
